# Predictors of Six-Month Functional Outcome After Chronic Subdural Hematoma Surgery: Logistic Regression Versus Machine Learning

**DOI:** 10.3390/jcm15176822

**Published:** 2026-09-03

**Authors:** Mirela Juković, Srdjan Stošić, Jagoš Golubović, Slobodan Pantelinac, Dejan B. Stojanović

**Affiliations:** 1 Faculty of Medicine, University of Novi Sad, Hajduk Veljkova 3, 21000 Novi Sad, Serbia; srdjan.stosic@mf.uns.ac.rs (S.S.); jagos.golubovic@mf.uns.ac.rs (J.G.); slobodan.pantelinac@mf.uns.ac.rs (S.P.); 2 Centre for Radiology, University Clinical Centre of Vojvodina, Hajduk Veljkova 1, 21000 Novi Sad, Serbia; 3 Department of Neurosurgery, University Clinical Centre of Vojvodina, Hajduk Veljkova 1, 21000 Novi Sad, Serbia; 4 Clinic of Physical Medicine and Rehabilitation, University Clinical Centre of Vojvodina, Hajduk Veljkova 1, 21000 Novi Sad, Serbia; 5 Institute of Lowland Forestry and Environment, University of Novi Sad, 21102 Novi Sad, Serbia; dejan.stojanovic@uns.ac.rs

**Keywords:** chronic subdural hematoma, Glasgow Outcome Scale, functional outcome, Karnofsky Performance Status, cortical atrophy grading, machine learning, random forest, XGBoost, multiple imputation

## Abstract

**Background/Objectives**: Six-month functional outcome after chronic subdural hematoma (CSDH) surgery has not been reliably predicted in Southeastern European cohorts. We asked whether machine learning (ML) classifiers outperform logistic regression. **Methods**: This study included a single-center retrospective cohort of 78 surgically treated CSDH patients (Novi Sad, Serbia). The primary outcome was favorable six-month outcome (Glasgow Outcome Scale 4–5). Missing covariates were multiply imputed (multiple imputation by chained equations, MICE, m = 20), and estimates were pooled using Rubin’s rules. A three-predictor model was pre-specified; six ML classifiers were trained on five preoperative predictors and evaluated using stratified 10-fold repeated cross-validation, with metrics derived from held-out predictions. **Results**: Favorable outcome occurred in 50/78 (64.1%). Preoperative Karnofsky Performance Status (KPS) was the only independent predictor (odds ratio, OR = 1.14 per point, 95% confidence interval, CI 1.07–1.20, *p* < 0.001). Cortical atrophy grade was associated univariately (OR = 0.57, 95% CI 0.34–0.96, *p* = 0.033) but not after adjustment for KPS (OR = 1.08, 95% CI 0.53–2.22, *p* = 0.833). Discrimination was indistinguishable across the six classifiers (AUC 0.870–0.885; all pairwise DeLong *p* ≥ 0.123); the three-predictor model reached an AUC of 0.903 (0.833–0.972). **Conclusions**: Preoperative KPS was the only independent predictor. No ML classifier outperformed logistic regression; a three-predictor model performed at least as well. External validation is required.

## 1. Introduction

Chronic subdural hematoma (CSDH) is among the most prevalent conditions encountered in neurosurgical practice. Population-based studies report overall incidence estimates spanning 3.4 to 39.1 per 100,000 person-years, with two Japanese series reporting 13.1 and 20.6 per 100,000 and rising to 58.1 per 100,000 in those aged 65 years or older and to 127.1 per 100,000 in those aged 80 years or older [1,2]. As global populations age, the epidemiological burden of CSDH is expected to rise substantially, placing increasing demands on neurosurgical services [3]. The condition disproportionately affects elderly individuals, many of whom carry significant comorbidities including anticoagulant use, cortical atrophy, and impaired cardiovascular reserve, factors that complicate both operative management and the anticipation of postoperative recovery. Current treatment of symptomatic CSDH is predominantly surgical. Burr hole craniostomy with closed-system drainage remains the most widely performed procedure and is associated with favorable outcomes in the majority of patients [4,5]. Several surgical techniques are in use; a network meta-analysis comparing burr hole with twist drill craniostomy found no difference in recurrence between single and double burr hole craniostomy [6]. Medical management with dexamethasone has been investigated, but patients who received dexamethasone had more adverse events than those given placebo [7]. Weigel et al. [8], in their systematic review, emphasized the value of closed-system drainage, which reduces the risk of recurrence without increasing the risk of infection.

Predicting which patients will achieve a favorable six-month functional outcome after surgical treatment of CSDH is clinically important for risk stratification, postoperative monitoring, rehabilitation, and identification of high-risk patients for intervention. Traditional approaches have relied on univariate analyses, simple multivariable logistic regression, or classification tree models incorporating readily available clinical and radiological variables such as preoperative Glasgow Coma Scale (GCS) score, patient age, hematoma thickness, hematoma density, midline shift, and the presence of cortical atrophy [9,10]. However, these factors do not operate in isolation. They may interact in ways that linear models do not always capture; whether accommodating such complexity yields materially better prediction in modest single-center cohorts, however, remains uncertain and is one of the questions this study addresses. In addition, most published prediction models were developed on small, homogeneous datasets with limited external validation.

Machine learning (ML) methods offer potential advantages over classical regression. Ensemble methods such as random forest and gradient boosting (XGBoost) can model complex non-linear interactions among predictors without requiring a priori variable selection or distributional assumptions [11,12]. Support vector machines and neural networks offer additional flexibility in representing high-dimensional decision boundaries. Several ML applications have been demonstrated in the management of brain and spine surgery, neuro-oncology, and radiology to predict outcome and postoperative functional state, including in cohorts of patients with different types of brain and spine lesions [13,14]. Systematic comparisons of multiple ML classifiers specifically for CSDH outcome prediction remain limited, and studies from Southeastern European centers, where patient demographics and surgical practices may differ from those of Western European or North American populations, are scarce.

In this study, we hypothesized that a compact set of preoperative clinical and radiological features would predict six-month functional outcome in CSDH patients and that ML classifiers would perform at least as well as traditional logistic regression. The specific aims were (1) to identify independent preoperative predictors using multiply imputed logistic regression on the full analytical cohort and a pre-specified parsimonious model; (2) to train and cross-validate six ML classifiers on a parsimonious feature set with out of fold performance estimation; and (3) to compare the discrimination and calibration of these classifiers against logistic regression in order to determine whether the additional complexity of machine learning is justified in a cohort of this size, treating the machine learning comparison as exploratory and hypothesis generating pending external validation.

## 2. Materials and Methods

### 2.1. Study Design and Setting

**Figure 1:**
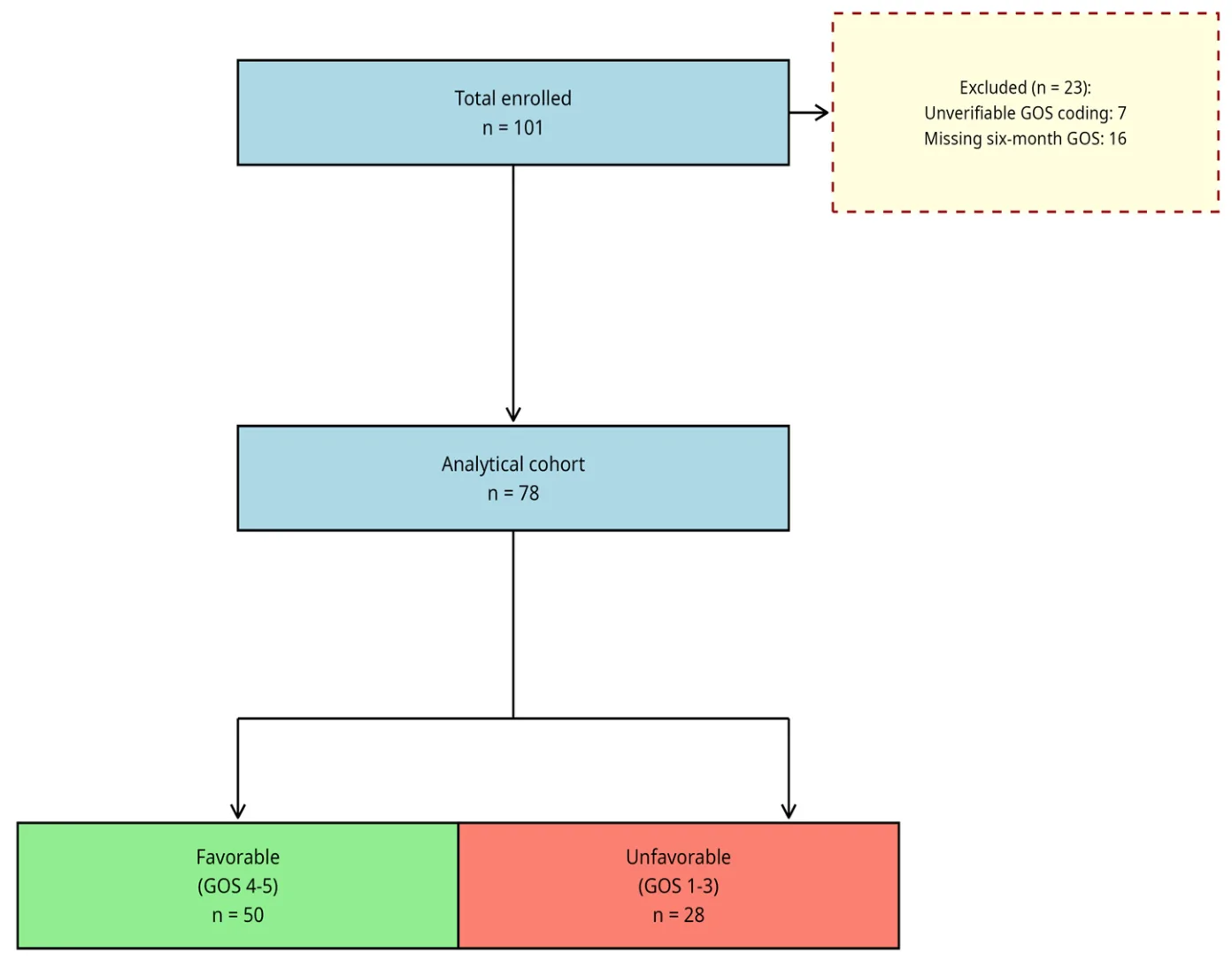
Patient flow diagram. Of 101 patients with CSDH managed at the neurosurgical unit during the study period, 23 were excluded (7 were excluded for unverifiable GOS coding and 16 for missing six-month GOS), leaving 78 patients in the analytical cohort. Favorable outcome at six months (GOS 4–5) were noted in 50 patients (64.1%); unfavorable (GOS 1–3) outcomes were noted in 28 patients (35.9%).

We conducted a single-center retrospective cohort study at the University Clinical Centre of Vojvodina, a tertiary referral hospital in Serbia over a three-year period. The cohort is historical and predates both the Dex-CSDH trial and the adoption of middle meningeal artery embolization. A total of 101 patients with CSDH managed at the neurosurgical unit during the study period were identified from the institutional registry, with no pre-analytical exclusions applied at the registry level; after application of the eligibility criteria specified below, 78 patients constituted the final analytical cohort (Figure 1).

### 2.2. Eligibility Criteria

Patients were eligible for inclusion if they were aged ≥18 years, had a CT-confirmed diagnosis of CSDH, and were managed (surgically or with initial conservative management followed by surgical drainage) at the index institution during the study period. CSDH was defined on CT scan as a crescentic extra-axial collection of blood products with CT density characteristics consistent with chronic or mixed-age blood (hypodense, isodense, or mixed-density collections). Subdural hygroma, predominantly CSF-containing collections without blood products, was distinguished from CSDH based on clinical history, prior neuroimaging, and CT characteristics. Patients were excluded if they had incomplete documentation precluding extraction of key predictor variables, lacked a six-month GOS assessment, or had a GOS status coded as ambiguous in the institutional database. All patients in the analytical cohort ultimately underwent surgical drainage, including those initially managed conservatively; the term “surgically treated” throughout refers to this final management.

### 2.3. Data Collection and Variables

Data were extracted from institutional medical records including operative notes, nursing documentation, discharge summaries, and outpatient follow-up records. Demographic and clinical variables included age (years), sex, GCS on admission (range 3–15), preoperative Karnofsky Performance Status (KPS; range 0–100), alcoholism, coagulopathy, diabetes mellitus, cardiac disease, and hepatic disease. Non-contrast computed tomography (CT) of the head was performed on a SOMATOM Sensation 64 multidetector CT scanner (Siemens Healthineers, Forchheim, Germany) using standard institutional protocol. Radiological variables included hematoma laterality (unilateral/bilateral), maximum hematoma width on CT (mm), CT density classification, and cortical atrophy. Cortical atrophy on CT scan was assessed using a four-point scale (grades 0–3) by one radiologist with five years of experience, following the visual grading described by Brugnara et al. [15]. Visual grading was rated as follows: grade 0, no cortical atrophy; grade 1, minor atrophy of the insular and/or the frontoparietal region, cerebral sulci slightly more pronounced; grade 2, moderate atrophy, further widening of the cerebral sulci; and grade 3, global atrophy, severely widened sulci, thinning of the cortical gyri. The grade was entered into all models as an ordinal score from 0 to 3, and a dichotomy (any atrophy, grades 1–3, versus none) was examined as a sensitivity analysis. All images were graded retrospectively, during revision of this manuscript, by a single reader in a dedicated re-read of the archived CT studies rather than from the routine radiology reports; the reader was not blinded to the six-month outcome. Inter-rater reliability could not be estimated, and no repeat reading was available for intra-rater reliability. Both issues are addressed in Section 4.7. CT density of the hematoma was classified into hyperdense, mixed, isodense, or hypodense patterns. Preoperative midline shift was recorded in millimeters in the CT report and analyzed as a continuous variable. Surgical variables included index management category (burr hole craniostomy, craniotomy, or initial conservative management followed by surgical drainage; no twist drill procedure was performed in this cohort), and use of closed-system subdural drainage. Outcome variables were GOS at six months (1–5), recurrence requiring re-operation, and in-hospital mortality. Anticoagulant and antiplatelet therapy were not available for systematic extraction and are discussed as a limitation.

### 2.4. Primary Outcome

The primary outcome was six-month functional status assessed using the Glasgow Outcome Scale (GOS) [16], dichotomized into favorable (GOS 4–5) versus unfavorable (GOS 1–3). GOS was ascertained from outpatient clinic notes and structured follow-up by the treating neurosurgeon; post-discharge mortality (GOS 1) was confirmed using official vital records. The score was assigned by the treating neurosurgeon during routine clinical follow-up rather than by an independent assessor, and the assessment was not blinded to the baseline clinical and imaging findings. No central adjudication of the outcome was undertaken. The resulting potential for outcome misclassification is discussed in Section 4.7.

### 2.5. Statistical Analysis

All analyses were performed in R version 4.4.0 (R Foundation for Statistical Computing, Vienna, Austria) [17] with random seed set to 2026. Key packages include mice v3.16.0 (multiple imputation and pooling) [18], caret v6.0-94 (ML training and cross-validation) [19], randomForest v4.7-1.1, XGBoost v1.7.6, e1071 v1.7-13 (SVM), nnet v7.3-19, rpart v4.1.23, and pROC v1.18.5 (area under the receiver operating characteristic curve, AUC, and DeLong’s test) [20].

Multiple imputation for missing covariates. Missing predictor values were handled using multiple imputation by chained equations (MICE) with m = 20 imputed datasets and 20 iterations per chain. Predictive mean matching was used for continuous variables, logistic regression for binary variables, and polytomous regression for surgery type. The outcome variable (dichotomized GOS) was not imputed.

Logistic regression. Univariate logistic regression was performed for each candidate predictor on each imputed dataset, with odds ratios (ORs) and 95% confidence intervals (CIs) pooled across the 20 imputations using Rubin’s rules [21]. Two multivariable logistic regression models were then fitted and pooled using Rubin’s rules across imputations: (a) a full adjusted model including age, sex, GCS, KPS, maximum hematoma width, surgery type, bilateral hematoma, drainage, cortical atrophy, and coagulopathy; and (b) a pre-specified parsimonious model including only KPS, cortical atrophy, and maximum hematoma width, three predictors chosen a priori on pathophysiological grounds to bring the events per variable (EPV) ratio to approximately 9 [22]. The association between the categorical CT density pattern and six-month GOS was assessed with the Kruskal–Wallis test, and the association between preoperative midline shift and GOS was assessed with Spearman’s rank correlation (Supplementary Figure S1).

Machine learning models. Six ML classifiers were trained on a parsimonious five-predictor feature set, KPS, cortical atrophy grade, maximum hematoma width, age, and preoperative GCS, which were selected on clinical grounds to constrain model complexity to the available data. The five-predictor set was fixed a priori on clinical and pathophysiological grounds and on the events per variable constraint (28 events). Candidate variables not included in the machine learning set (laterality, CT density, midline shift, coagulopathy, surgery type, drainage) were either non-significant on univariate screening or produced quasi-separation. Male sex is a deliberate exception. It was significantly associated with outcome univariately (OR = 4.09, 95% CI 1.51–11.12, *p* = 0.006) but was omitted because it is neither modifiable nor mechanistically interpretable as a preoperative target and because the events per variable constraint permitted only five terms. Its exclusion is a design choice, not a screening result. Classifiers included (1) logistic regression; (2) random forest (500 trees, mtry = 2); (3) XGBoost (50 rounds, max_depth = 3, eta = 0.1, subsample = 0.8, colsample_bytree = 0.8); (4) radial kernel support vector machine (SVM; cost = 1, gamma = 1/*p*); (5) single hidden layer neural network (size = 3, decay = 0.1); and (6) classification and regression tree (CART, complexity parameter selected by the internal 10-fold cross-validation of the training fold). Hyperparameters other than the CART complexity parameter were fixed a priori rather than tuned, so that no tuning information could leak across folds, and all six classifiers were evaluated on identical cross-validation folds. Evaluation used stratified 10-fold repeated cross-validation (5 repeats = 50 held-out fold evaluations per model). For the machine learning analyses, missing predictor values were completed with a single MICE imputation (predictive mean matching for all five predictors, none of which is binary) fitted on the full analytical cohort (*n* = 78), including the outcome variable, before cross-validation rather than re-estimated within each training fold, so that all 78 cases were retained. Because this imputation was not fold internal, a small optimistic bias in the discrimination estimates cannot be fully excluded, although it is expected to be minor given the low missingness within the analytical cohort itself (0–2.6% across the five predictors: age 0%, preoperative GCS 1.3%, preoperative KPS 1.3%, maximum hematoma width 2.6%, cortical atrophy grade 0%; Supplementary Table S1). All reported performance metrics (AUC, sensitivity, specificity, positive and negative predictive values, accuracy, and Brier score) were computed from out of fold predictions aggregated across repeats, which removes the in-sample optimism arising from model fitting; the full set of metrics, including PPV and NPV, is reported in Supplementary Table S2. For each subject the held-out predicted probability was the mean of the five cross-validated predictions in which the subject was in the test fold; this aggregated probability was used for receiver operating characteristic (ROC) analysis, confusion matrix-based metrics (at a 0.50 threshold), and the Brier score [23]. Pairwise model comparisons used DeLong’s test for correlated ROC curves [24].

Sensitivity analyses. Sensitivity analyses included comparison of complete case and multiply imputed regression estimates, subgroup analysis by sex and age, and model comparison using the pre-specified three-predictor inferential model against the five-predictor classifier set, both put through the identical cross-validation pipeline. An ordinal proportional odds model for full GOS 1–5 was reported as a complementary analysis (Supplementary Table S3). A KPS-excluded analysis was also performed, in which all six classifiers were re-trained on the four remaining preoperative predictors (age, preoperative GCS, cortical atrophy, and maximum hematoma width) under the identical stratified 10-fold repeated cross-validation pipeline (Supplementary Table S4). Decision curve analysis [25] was performed on the out of fold predicted probabilities of the five-predictor logistic classifier and XGBoost, comparing net benefit against the treat all and treat none reference strategies across the range of threshold probabilities (Supplementary Figure S2).

Penalized regression. The full ten-term multivariable model produced quasi-separation in the individual imputed datasets because no patient with coagulopathy achieved a favorable outcome, and its events per variable ratio was 2.5 (28 events for 11 estimated coefficients). It was therefore re-estimated using the penalized-likelihood method of Firth [26,27], which yields finite estimates under separation, and pooled across the 20 imputations by Rubin’s rules. Ridge and lasso logistic regression, with the penalty selected by 10-fold cross-validation within each imputed dataset, were fitted as further shrinkage checks [28]. The full model is reported as exploratory throughout; the pre-specified parsimonious model remains the primary inferential result. Collinearity between cortical atrophy grade and age was examined using Spearman correlation, using variance inflation factors and by adding age to the parsimonious model.

Attrition and missing outcomes. Included (*n* = 78) and excluded (*n* = 23) patients were compared on every variable recorded in the registry, using the Wilcoxon rank-sum test for continuous variables and Fisher’s exact test for binary variables (Supplementary Table S5). Three further analyses addressed the missing six-month outcomes. First, predictors were multiply imputed on the full registry (*n* = 101; the outcome was never imputed). A logistic model for the probability of inclusion was fitted, and the parsimonious outcome model was refitted on the analytical cohort with stabilized inverse probability of inclusion weights [29]. Second, two extreme case scenarios assigned a favorable, and then an unfavorable, outcome to all 23 excluded patients. Third, a pattern mixture analysis drew the outcome of the excluded patients from the fitted model with the log-odds shifted by a constant delta ranging from −3 to +2. Results are given in Supplementary Table S8.

## 3. Results

### 3.1. Patient Flow and Exclusions

Of 101 patients with CSDH managed at the neurosurgical unit during the study period, 23 were excluded prior to analysis: 7 with unverifiable GOS coding and 16 with no six-month GOS recorded. The final analytical cohort comprised 78 patients (Figure 1). Multiple imputation retained all 78 cases for regression analyses. Included and excluded patients differed on only one recorded variable, documented drain use (Supplementary Table S5).

Included and excluded patients were compared on every variable available in the registry (Supplementary Table S5). With one exception, no comparison reached significance: age 69.1 ± 11.4 versus 72.6 ± 14.5 years (*p* = 0.12), preoperative GCS 12.8 ± 2.9 versus 14.1 ± 1.4 (*p* = 0.16), preoperative KPS 52.2 ± 20.3 versus 64.3 ± 17.2 (*p* = 0.16), maximum hematoma width 22.1 ± 8.0 versus 25.6 ± 6.6 mm (*p* = 0.22), male sex 66.7% versus 56.5% (*p* = 0.46), and recurrence 17.9% versus 18.2% (*p* = 1.00). The exception was recorded drain use (96.6% versus 72.7%, *p* = 0.026), a difference that rests on the 11 excluded patients in whom the variable was documented at all and that therefore concerns documentation rather than management. Cortical atrophy could not be compared because CT studies were only available for grading of only one excluded patient. The comparison is further limited by missingness since 14 to 16 of the 23 excluded patients lacked the clinical variables being compared. Thus, most tests rest on 7 to 9 excluded cases and are correspondingly underpowered. The direction of the differences is nevertheless informative. Excluded patients tended to present with higher GCS and higher KPS, which is consistent with better functioning patients not returning for six-month review and would, if anything, bias the analytical cohort toward a worse rather than a more favorable case mix.

### 3.2. Baseline Characteristics

**Figure 2:**
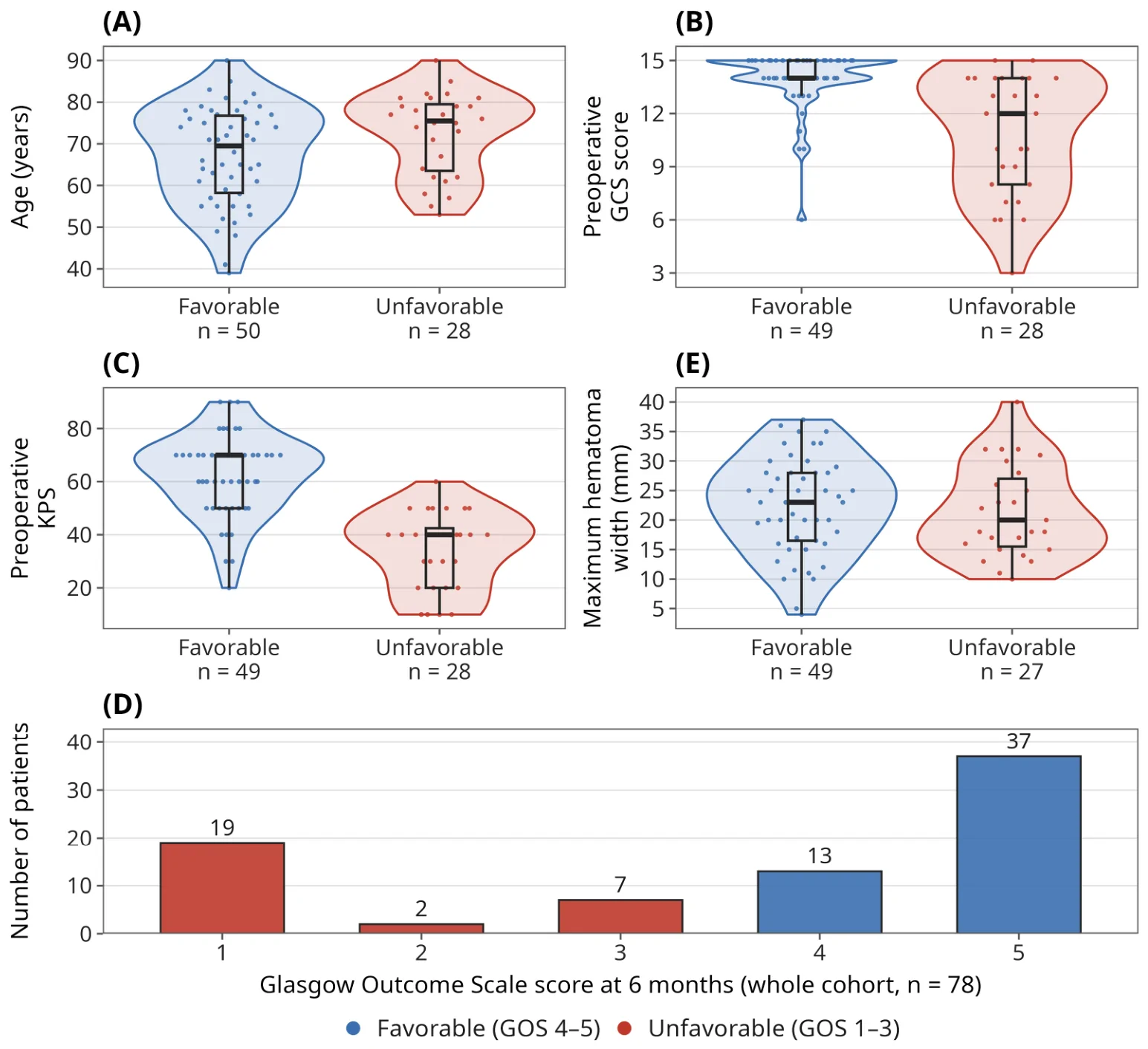
Preoperative predictors and six-month outcome distribution in the analytical cohort (*n* = 78). (**A**) Age (*n* = 78), (**B**) preoperative Glasgow Coma Scale score (*n* = 77), (**C**) preoperative Karnofsky Performance Score (*n* = 77), and (**E**) maximum hematoma width (*n* = 76) are shown as violin plots overlaid with box plots and individual patient jitter, stratified by favorable (blue) versus unfavorable (red) six-month outcome; the numbers differ because of missing values, which were not imputed for this figure. Panel (**D**) shows the overall distribution of the six-month Glasgow Outcome Scale across the cohort (GOS 1, *n* = 19; GOS 2, *n* = 2; GOS 3, *n* = 7; GOS 4, *n* = 13; GOS 5, *n* = 37).

Baseline characteristics of the 78 included patients are summarized in Table 1. The mean age was 69.1 ± 11.4 years (median 72, range 39–90); 52/78 (66.7%) were male. Median GCS on admission was 14 (IQR 12–15). Mean preoperative KPS was 52.2 ± 20.3. Recurrence requiring re-operation occurred in 14/78 (17.9%); in-hospital mortality occurred in 4 patients (5.1% of the cohort; the variable was not documented for one patient). Cortical atrophy grade was 0 in 11 patients (14.1%), 1 in 18 (23.1%), 2 in 29 (37.2%), and 3 in 20 (25.6%). The distribution of key continuous predictors by outcome group is shown in Figure 2.

### 3.3. Outcome Distribution

At six months, favorable outcome (GOS 4–5) was recorded in 50/78 patients (64.1%), and unfavorable outcome (GOS 1–3) was recorded in 28/78 (35.9%). GOS grades were as follows: 5 (good recovery) in 37 (47.4%), 4 (moderate disability) in 13 (16.7%), 3 (severe disability) in 7 (9.0%), 2 (vegetative state) in 2 (2.6%), 1 (death) in 19 (24.4%). Of the 19 deaths, 4 occurred in hospital and 15 after discharge, reflecting the elderly, low-functional-reserve case mix of this subgroup. The 19 patients who died had a mean age of 73.6 years (SD 10.1) and a mean preoperative KPS of 32.1 (SD 15.1), against 69.1 years and 52.2, respectively, for the cohort as a whole.

### 3.4. Univariate Logistic Regression

Univariate logistic regression on the 20 imputed datasets (Rubin’s rules pooling, *n* = 78) identified preoperative KPS as the strongest individual predictor of favorable outcome (pooled OR = 1.12 per point, 95% CI 1.07–1.19, *p* < 0.001), followed by GCS on admission (OR = 1.66 per point, 95% CI 1.28–2.15, *p* < 0.001) and male sex (OR = 4.09, 95% CI 1.51–11.12, *p* = 0.006). Cortical atrophy grade was also associated with outcome, each additional grade reducing the odds of a favorable result by about 40% (OR = 0.57, 95% CI 0.34–0.96, *p* = 0.033). The dichotomy of any atrophy versus none was not significant (OR = 0.35, 95% CI 0.07–1.75, *p* = 0.20), which is unsurprising given that only 11 of the 78 patients had no atrophy at all. Age was weakly associated (OR = 0.96 per year, 95% CI 0.913–0.999, *p* = 0.047). CT density pattern was not associated with six-month outcome (Kruskal-Wallis H = 1.97, df = 3, *p* = 0.58), and preoperative midline shift did not predict GOS (per-mm OR = 1.02, 95% CI 0.94–1.10, *p* = 0.68; Spearman rho = 0.14, *p* = 0.21). These null imaging associations are shown in Supplementary Figure S1.

### 3.5. Multivariable Logistic Regression

**Figure 3:**
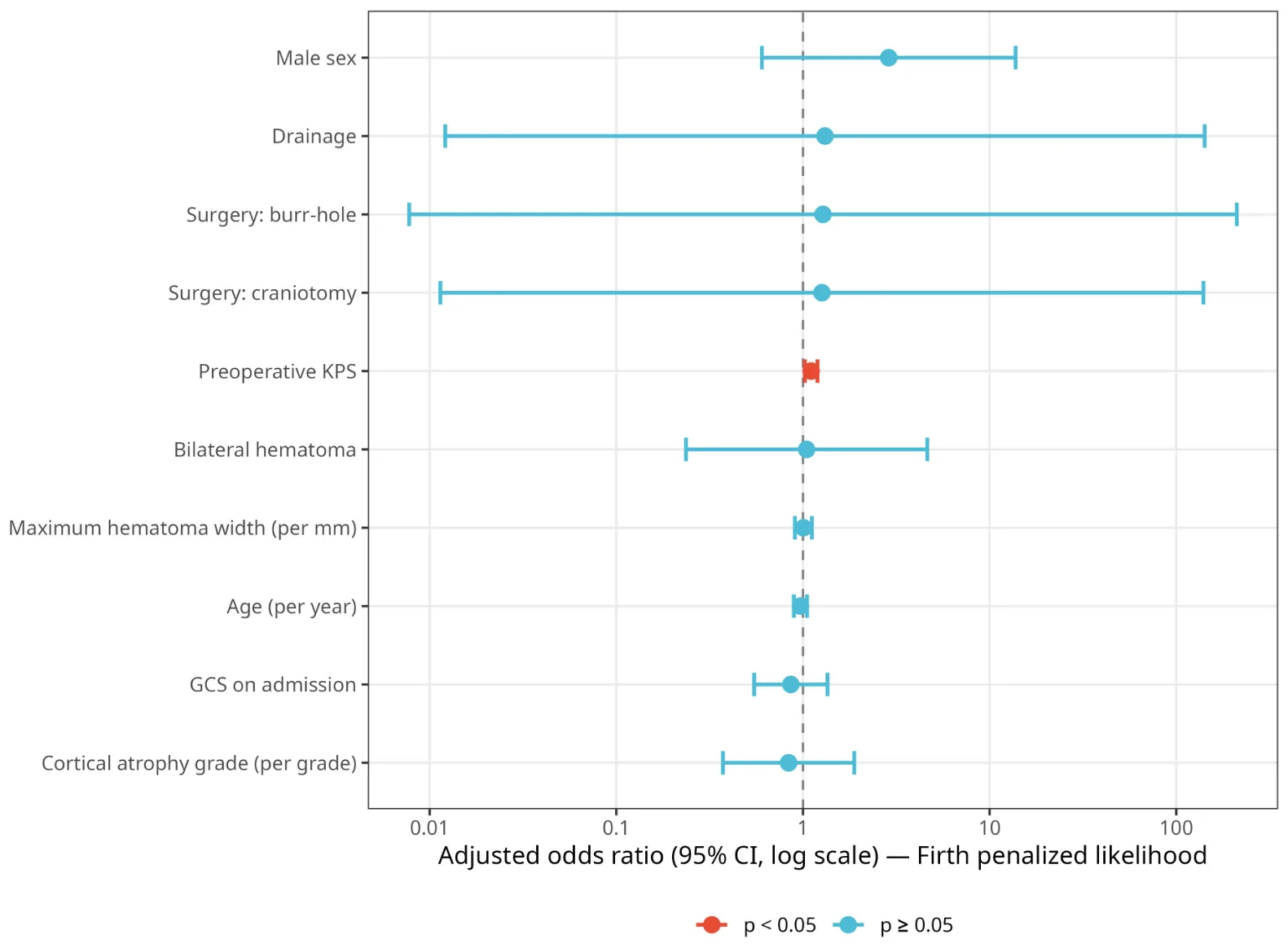
Forest plot of adjusted odds ratios from the exploratory full multivariable model, estimated using Firth penalized likelihood (*n* = 78; m = 20 imputations, Rubin’s rules pooling). Odds ratios are shown with 95% confidence intervals on a log scale. The vertical dashed line marks an odds ratio of 1 (no effect) and intervals that cross it are compatible with no association. Cortical atrophy was entered as the four-point visual grade. No patient with coagulopathy had a favorable outcome (0 of 9), so its penalized estimate is a function of that zero cell rather than an effect size on the same scale as the other terms. It is reported in Table 2 and Supplementary Table S6.

The full ten-term multivariable model is reported as an exploratory analysis only. Its events per variable ratio was 2.5 (28 events for 11 estimated coefficients) and unpenalized maximum likelihood produced quasi-separation because no patient with coagulopathy achieved a favorable outcome (0 of 9). Thus, the coagulopathy, drainage, and surgery type coefficients diverged. Re-estimated by Firth penalized likelihood and pooled over the 20 imputations (upper panel of Table 2 and Supplementary Table S6), the model returned finite estimates for every term. Preoperative KPS remained independently associated with favorable outcome (OR = 1.11 per point, 95% CI 1.02–1.20, *p* = 0.011). Coagulopathy, the term responsible for the separation, returned a nominally significant penalized estimate (OR = 0.03, 95% CI 0.001–0.64, *p* = 0.025), but this rests entirely on the absence of any favorable outcome among the nine patients so classified and cannot be read as an effect estimate. For that reason, it is omitted from Figure 3. Cortical atrophy grade was not associated with outcome (OR = 0.84, 95% CI 0.37–1.88, *p* = 0.667). In the pre-specified parsimonious model (lower panel of Table 2, EPV ≈ 9), preoperative KPS was again the only independent predictor (OR = 1.14 per point, 95% CI 1.07–1.20, *p* < 0.001). The effect of cortical atrophy grade seen on univariate analysis did not survive adjustment for KPS (OR = 1.08, 95% CI 0.53–2.22, *p* = 0.833), and maximum hematoma width remained non-significant (OR = 1.08, 95% CI 0.98–1.19, *p* = 0.126). Lasso regression assigned non-zero coefficients in all 20 imputed datasets to preoperative KPS, coagulopathy, sex, and cortical atrophy grade, but the atrophy coefficient was shrunk to −0.008 (OR 0.99), that is to effectively nothing. Ridge regression did not simply shrink the unpenalized solution. Three coefficients changed signs. The atrophy coefficient increased in absolute value (−0.177 to −0.234), and atrophy rose from sixth to third in coefficient magnitude, which shows that the ranking of terms in the full model is not stable under penalization (Supplementary Table S7). Adjusted odds ratios from the Firth penalized model are visualized in Figure 3. Cortical atrophy grade rose steeply with age (mean age 53.4 years at grade 0, 66.9 at grade 1, 72.0 at grade 2 and 75.7 at grade 3; Spearman rho = 0.54, *p* < 0.001; +6.7 years per grade, 95% CI 4.6–8.8), but collinearity was not the explanation for its loss of significance. The highest variance inflation factor in a seven-term complete case clinical model (*n* = 75) was 2.68 (for KPS), and all others were 2.54 or lower. Adding age to the parsimonious model left the atrophy coefficient near unity (OR = 0.97, 95% CI 0.42–2.22, *p* = 0.940), while age itself was not significant (OR = 1.02, 95% CI 0.94–1.11, *p* = 0.601).

### 3.6. Machine Learning Model Performance

**Figure 4:**
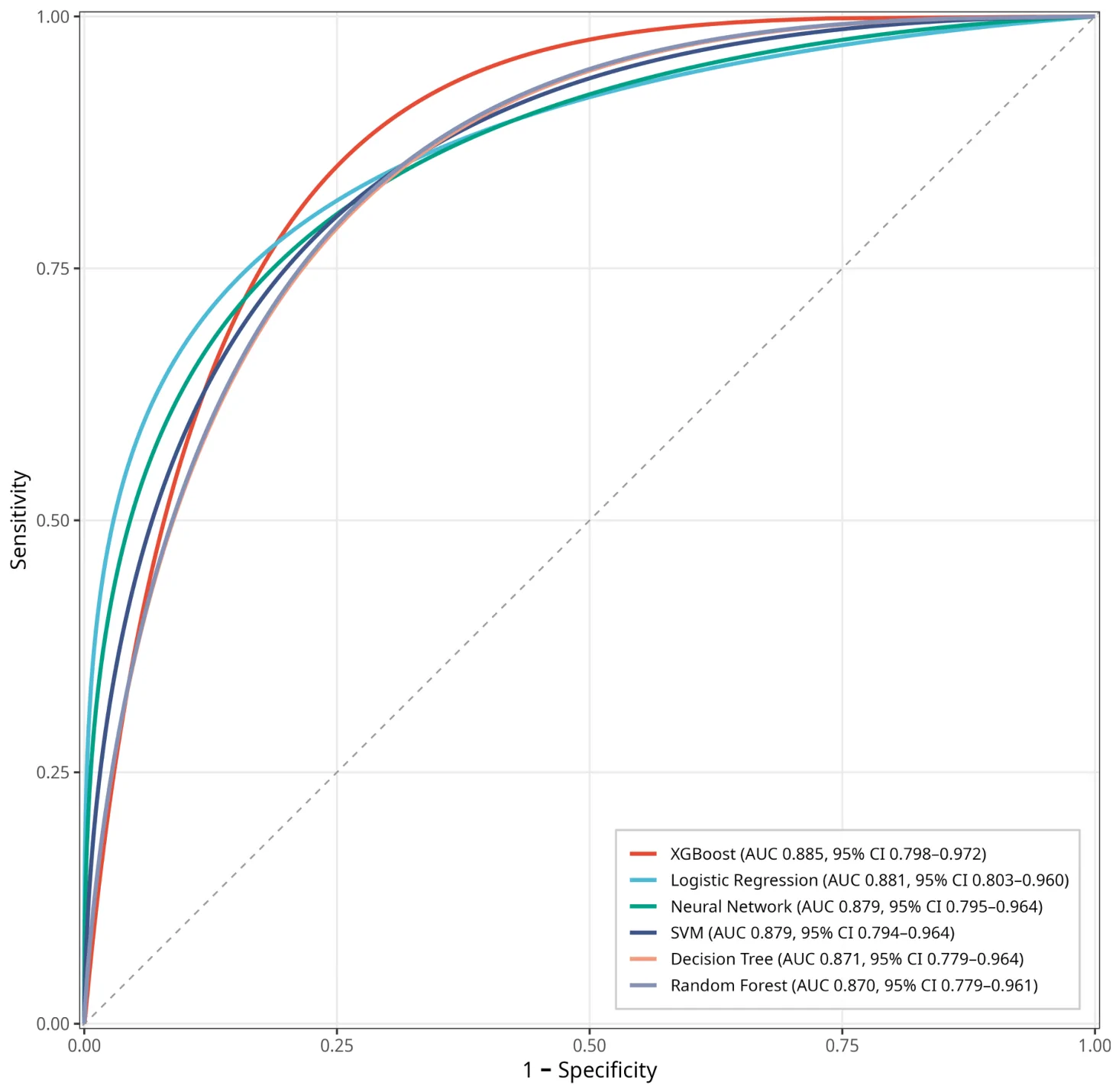
Receiver operating characteristic curves for all six machine learning classifiers. The dashed diagonal marks chance performance (AUC = 0.5). Curves are derived exclusively from held-out predictions aggregated across stratified 10-fold repeated cross-validation (5 repeats) and are drawn with a binormal parametric fit for legibility; the areas under the curve and their DeLong 95% confidence intervals shown in the legend are those of the underlying empirical curves, not of the fitted ones.

Performance metrics for all six ML classifiers evaluated using stratified 10-fold repeated cross-validation (5 repeats, all metrics from held-out fold predictions) are presented in Table 3 and visualized as ROC curves in Figure 4.

XGBoost demonstrated the most balanced performance: the highest AUC (0.885), the lowest Brier score (0.120), and, together with logistic regression and the decision tree, the highest accuracy (0.833). Pairwise DeLong’s test showed no statistically significant AUC difference between any two classifiers. The smallest *p* value across all fifteen comparisons was 0.123 (XGBoost versus random forest), and all others exceeded 0.37. The entire spread between the best and the worst classifier was 0.015 in AUC. Confidence intervals overlap almost completely, consistent with the modest sample size.

All reported metrics are derived exclusively from out of fold held-out predictions, which removes the optimism that arises from evaluating a model on the data used to fit it; two residual sources of optimism remain. First, the imputation used to complete missing predictors was fitted once on the full analytical dataset before cross-validation rather than within each fold. Second, the five-predictor feature set was chosen after univariate screening on the complete analytical cohort rather than inside each training fold, so predictor selection, like the imputation, was not fold internal. A small upward bias in the discrimination estimates therefore cannot be fully excluded (see Section 2 and Supplementary Table S1).

The pre-specified three-predictor inferential model (preoperative KPS, cortical atrophy grade, and maximum hematoma width) was put through the identical cross-validation pipeline so that its discrimination could be compared with that of the five-predictor classifier set on the same folds. Its held-out discrimination was AUC 0.903 (95% CI 0.833–0.972), with a sensitivity of 0.88, specificity of 0.68, accuracy of 0.808, and a Brier score of 0.125. The difference from the five-predictor logistic classifier (AUC 0.881) was 0.021 in favor of the smaller model and did not reach significance (DeLong *p* = 0.104). Calibration was also closer to ideal. The calibration slope of the three-predictor model was 0.83 (95% CI 0.47–1.19), consistent with unity, whereas that of the five-predictor logistic classifier was 0.69 (95% CI 0.38–0.99), whose upper bound excludes unity and indicates measurable over-fitting. Calibration in the large was close to zero for both (−0.03 and −0.05 on the log-odds scale). Adding age and preoperative GCS to the three clinical predictors therefore bought no discrimination and cost calibration.

### 3.7. Variable Importance

**Figure 5:**
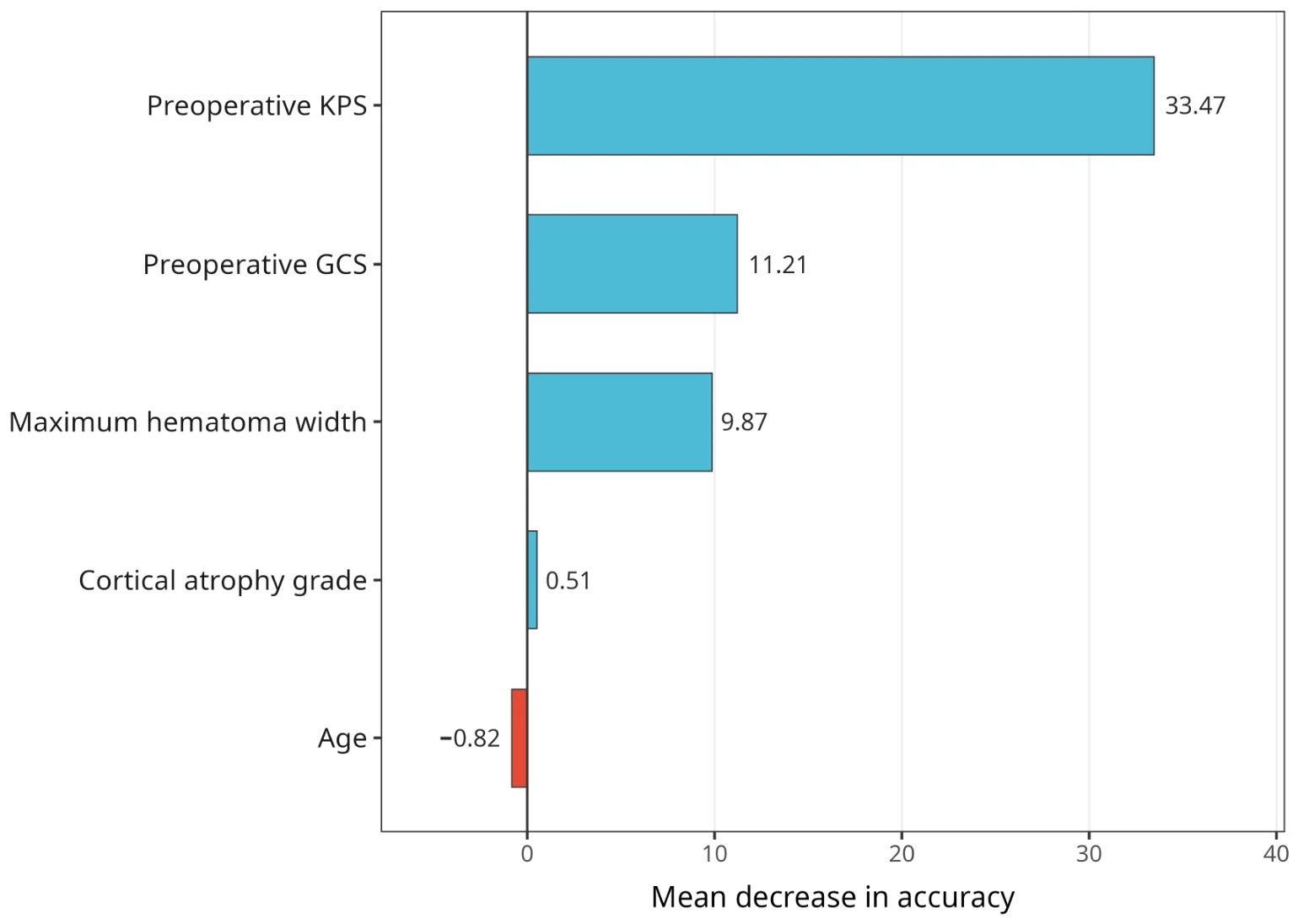
Variable importance from the random forest classifier (mean decrease in accuracy). Variables are reported in descending order of importance: preoperative Karnofsky Performance Score (33.5), preoperative GCS (11.2), maximum hematoma width (9.9), cortical atrophy grade (0.5), and age (−0.8). Blue bars mark positive importance, the red bar has a negative value. A negative value means that permuting the variable did not reduce out of bag accuracy.

Random forest variable importance (mean decrease in accuracy; Figure 5) ranked predictors as follows: (1) preoperative KPS (33.5), (2) preoperative GCS (11.2), (3) maximum hematoma width (9.9), (4) cortical atrophy grade (0.5), and (5) age (−0.8). KPS dominated the ranking by a factor of three over the next predictor, consistent with the univariate and parsimonious multivariable regression findings. The negative value for age indicates that permuting it did not degrade out of bag accuracy at all.

### 3.8. Sensitivity Analyses

The ordinal proportional odds model on the full GOS scale (Supplementary Table S3) reproduced the parsimonious result. Specifically, preoperative KPS remained strongly associated with a better outcome (OR = 1.11 per point, 95% CI 1.07–1.15, *p* < 0.001), while cortical atrophy grade did not (OR = 0.94, 95% CI 0.56–1.58, *p* = 0.82). Three further analyses addressed the 23 excluded patients, 16 of whom had no six-month outcome recorded and 7 of whom had GOS coding that could not be verified (Supplementary Table S8). Weighting the analytical cohort by the stabilized inverse probability of inclusion (weights 0.78 to 2.30, median 0.96) reproduced the primary estimates almost exactly (KPS OR = 1.14, 95% CI 1.07–1.21, *p* < 0.001; cortical atrophy grade OR = 1.08, 95% CI 0.52–2.24, *p* = 0.84). Assigning a favorable outcome to all 23 excluded patients left KPS significant (OR = 1.10, 95% CI 1.05–1.15, *p* < 0.001), as did assigning an unfavorable outcome to all 23 (OR = 1.07, 95% CI 1.03–1.10, *p* < 0.001); both scenarios are deliberately implausible bounds. In the pattern mixture analysis, the KPS effect remained significant across the entire range examined (delta from −3 to +2: OR 1.10 to 1.14, all *p* < 0.001), and cortical atrophy grade remained non-significant throughout (all *p* ≥ 0.71). The conclusions are therefore not contingent on the missing at random assumption.

### 3.9. Secondary Analysis: Recurrence

Recurrence requiring re-operation occurred in 14/78 (17.9%). On univariate analysis the only preoperative variable associated with recurrence was a lower preoperative Karnofsky score (OR = 0.96 per point, 95% CI 0.93–0.99, *p* = 0.008). A higher cortical atrophy grade (OR = 1.95 per grade, 95% CI 0.98–3.87, *p* = 0.058) and a lower GCS on admission (OR = 0.84 per point, 95% CI 0.70–1.01, *p* = 0.060) approached but did not reach significance. Bilateral hematoma was not associated with recurrence (11 of 50 unilateral versus 3 of 28 bilateral hematomas recurred; OR = 0.43, 95% CI 0.11–1.68, *p* = 0.22). Drain use could not be assessed because only two of the 58 patients in whom the variable was documented were managed without a drain and neither recurred. With 14 events, multivariable modeling of recurrence is not feasible (fewer than five events per candidate variable) and is recommended as a priority for future larger studies.

## 4. Discussion

### 4.1. Summary of Main Findings

In this methodologically revised analysis of a single-center retrospective cohort of 78 CSDH patients, preoperative Karnofsky Performance Score was the only independent predictor of six-month functional outcome in a pre-specified three-predictor logistic regression model (EPV ≈ 9) fitted on the full cohort using multiple imputation and Rubin’s rules pooling. Cortical atrophy, graded on a four-point visual scale, was associated with outcome on univariate analysis but not after adjustment for preoperative functional status. Machine learning classifiers trained on a compact five-predictor feature set and evaluated entirely by held-out repeated cross-validation yielded discrimination in the 0.870–0.885 AUC range, with no statistically significant pairwise difference between any two classifiers. XGBoost had the numerically highest AUC and the best calibration, and logistic regression was 0.004 lower in AUC, a difference far smaller than this cohort could detect. The machine learning comparison is therefore exploratory and hypothesis generating rather than evidence that any algorithm is superior, and external validation remains mandatory. Because every reported metric derives from predictions made on data not seen during model training, the estimates are largely free of in-sample optimism. The principal residual caveats are that missing predictors were imputed once on the full dataset before cross-validation and that the feature set was selected outside the cross-validation loop, so a small optimistic bias cannot be entirely excluded.

### 4.2. Cortical Atrophy and Functional Outcome

Familiari et al. [30] proposed that cortical atrophy is a triggering factor in CSDH formation, with the enlarged subdural space favoring membrane formation, activation of the inflammatory cascade, trans-endothelial cellular filtration, and neoangiogenesis. Abouzari et al. [10] included brain atrophy among the predictors of poor outcome in their regression model. In the present cohort, the picture is more qualified. Cortical atrophy grade was indeed related to outcome when considered alone, with each additional grade reducing the odds by about 40% (OR = 0.57 per grade, 95% CI 0.34–0.96, *p* = 0.033), and grade 3 was recorded in 11 of the 28 patients with an unfavorable outcome (39.3%) but in only 9 of the 50 with a favorable one (18.0%). That association did not survive adjustment for preoperative Karnofsky score, in the parsimonious model, in the Firth penalized full model, in the ordinal model on the full GOS scale, or under inverse probability weighting for attrition. In addition, lasso shrank the atrophy coefficient to −0.008 (OR 0.99), effectively nothing. Atrophy grade rose steeply with age (Spearman rho = 0.54, *p* < 0.001; +6.7 years per grade), yet adding age to the model did not restore the effect. The highest variance inflation factor in the seven-term complete case clinical model was 2.68, so simple collinearity with age is not the explanation. The more likely reading is that cortical atrophy is a marker of the frailty that the Karnofsky score already captures more directly. Because grading was performed by a single reader who was not blinded to the outcome, reproducibility could not be quantified, and a volumetric measure read blind to outcome by two or more readers is the obvious next step. One further observation belongs here. Although atrophy grade carried no independent association with six-month function, it was the variable most nearly associated with re-operation in this cohort (OR = 1.95 per grade, 95% CI 0.98–3.87, *p* = 0.058), while preoperative KPS predicted recurrence as well (OR = 0.96 per point, *p* = 0.008). Atrophy may therefore be a predictor of recurrence rather than of functional outcome, which would reconcile the present result with the recurrence literature. With 14 events, this cohort cannot settle the question, and it is a specific priority for larger series.

### 4.3. Preoperative Karnofsky Performance Score

KPS emerged as the top predictor across all analytical frameworks (univariate, multiply imputed adjusted, parsimonious, and RF variable importance). KPS is a well-validated, clinician-rated measure of functional capacity that captures information about pre-morbid health status not fully reflected in neurological examination scores alone. Its practical advantage lies in its clinical ubiquity. It is a brief, widely used clinician-rated scale [31]. In addition, where it is already part of the preoperative assessment, it is immediately deployable at no additional cost in a clinical prediction tool without additional resource requirements [31].

Because KPS is a clinician-rated measure of global functional capacity, it is conceptually proximal to the functional outcome endpoint (GOS). Part of its predictive strength may therefore reflect baseline functional status rather than an independent, mechanistically distinct prognostic signal. Consistent with this concern, a sensitivity analysis excluding KPS was performed (Supplementary Table S4). Removing KPS lowered discrimination substantially (logistic regression AUC 0.881 to 0.779; XGBoost 0.885 to 0.726; random forest 0.870 to 0.723), and the leading model retained only moderate discrimination (logistic regression AUC 0.779; range across the six classifiers 0.723–0.779). The remaining preoperative variables—age, preoperative GCS, cortical atrophy grade, and maximum hematoma width—therefore carry some independent information, but a drop of 0.10 to 0.16 in AUC shows that most of the discrimination in these models comes from KPS. KPS should thus be interpreted primarily as a strong marker of premorbid functional status rather than a modifiable therapeutic target.

### 4.4. Comparison with Published Machine Learning Studies in CSDH

Several recent studies have applied ML methods to CSDH. Fuse et al. [32] trained four algorithms (logistic regression, support vector machine, random forest, and light gradient boosting machine) on preoperative data from one institution (*n* = 188) and validated them at a second institution (*n* = 99), predicting an unfavorable functional outcome (modified Rankin Scale 3–6) at hospital discharge. Discrimination was high in both settings (internal ROC-AUC 0.906–0.925, external 0.833–0.860) [32]. Ni et al. [33] addressed a different endpoint, recurrence within three months of surgery, in 447 patients using eight algorithms with synthetic minority over-sampling. Their best model reached an AUC of 0.928 in the development cohort but 0.834 in the held-out test cohort, a gap that illustrates how much apparent performance can depend on the evaluation set [33]. The present study differs from both in targeting six-month GOS as a patient-centered outcome and in comparing six classifiers head-to-head on identical cross-validation folds. Unlike Fuse et al., [32] however, it has no external validation cohort, which remains the principal limitation relative to that work.

### 4.5. Logistic Regression Versus Machine Learning Classifiers

On this five-predictor set, logistic regression (AUC 0.881) was within 0.004 of the best ensemble method (XGBoost 0.885) and 0.011 above random forest (0.870), and no pairwise DeLong comparison was significant. The almost completely overlapping confidence intervals indicate that the apparent differences reflect sampling variability rather than true algorithm superiority. This absence of a detected difference must not be read as evidence of equivalence. With 50 favorable and 28 unfavorable outcomes, and taking the standard errors implied by the reported DeLong intervals together with the observed correlation between the classifiers’ predictions (Pearson r 0.87 to 0.97, median 0.93), the study had 80% power to detect an AUC difference of about 0.031 between the best and the worst classifier. The entire observed spread was 0.015, that is about 47% of the smallest difference the study could have detected. The comparison is therefore underpowered by construction: this cohort could not have distinguished these algorithms even if they genuinely differed. The machine learning comparison should therefore be regarded as exploratory and hypothesis generating. Because logistic regression matched the more complex algorithms while remaining simpler, transparent, and directly interpretable, a logistic risk score is the preferable option for routine clinical use, with ensemble methods reserved for research settings or richer feature sets (radiomics, biomarkers). Although XGBoost did not produce a statistically higher AUC than logistic regression or random forest (DeLong test versus logistic regression *p* = 0.901; versus random forest *p* = 0.123), it produced the lowest Brier score (0.120) and a balanced sensitivity/specificity profile (0.86/0.79). The Brier score is an overall measure of probability accuracy that combines discrimination and calibration rather than a calibration measure as such. On the calibration slope, XGBoost was indeed close to ideal (0.94, 95% CI 0.56–1.32). Although calibration and class balance are clinically relevant, these marginal gains do not by themselves justify a more complex model. A well-calibrated logistic score can be obtained using standard recalibration, so the simpler, interpretable logistic model remains the preferred basis for any future decision-support tool.

The similarity of logistic regression AUC to ensemble methods is a notable finding: on this five-feature parsimonious set, the outcome surface is adequately captured by a linear log-odds model, and the addition of non-linear interactions contributes little further discrimination.

### 4.6. Clinical Utility and Decision Support

A practical clinical prediction tool derived from the three-predictor logistic model could be implemented at the preoperative stage to generate individualized probability estimates for six-month GOS ≥ 4. Such a tool would require only three variables collected in routine clinical practice: preoperative KPS, CT-based maximum hematoma width, and the visual cortical atrophy grade. Of these, only KPS carried an independent effect in this cohort. The pooled coefficients of that model are given in Table 4 so that the predicted probability can be computed for an individual patient and the model can be validated externally by other groups. These are apparent estimates. No uniform shrinkage factor was applied (the heuristic value implied at *n* = 78 with three predictors is 0.94), and the bootstrap-corrected apparent C-statistic of the model fitted on the whole cohort was 0.907 (optimism 0.012, B = 500). They will over-fit somewhat when applied to new patients and should be recalibrated before any external use. The data collection era predates the Dex-CSDH trial [7] and the emergence of middle meningeal artery embolization; prospective external validation in a contemporary cohort is required before clinical deployment. Decision curve analysis of the out of fold predicted probabilities of the five-predictor logistic classifier and XGBoost (Supplementary Figure S2) showed net benefit over both the treat all and treat none strategies across threshold probabilities from approximately 0.24 to 0.93 (outcome prevalence 0.641). Below a threshold of about 0.24, the treat all strategy was equal or superior, as expected at this prevalence; however, because this analysis rests on internally cross-validated predictions from a single small center, external validation in an independent, contemporary cohort remains mandatory before any clinical deployment.

### 4.7. Limitations

Several limitations follow from the design. This was a retrospective study conducted at a single institution in Serbia. Case mix, surgical technique preferences, postoperative management protocols, and follow-up practices all vary across centers, so the findings may not be generalized. The retrospective design also leaves room for information bias and unmeasured confounding. A further concern is attrition. In total, 23 of the 101 registry patients (22.8%) were excluded, including 16 because no six-month GOS was recorded and 7 because the recorded GOS coding could not be verified. Deaths were not a reason for exclusion; the 19 patients who had died by six months were retained and coded GOS 1. Patients lost to follow-up may nevertheless differ systematically from those retained, so selection bias cannot be excluded. Included and excluded patients were therefore compared on every variable recorded in the registry, with the sole exception of documented drain use (96.6% versus 72.7%, *p* = 0.026), a difference that concerns documentation rather than management (Section 3.1 and Supplementary Table S5). In addition, the influence of the missing outcomes was quantified using inverse probability weighting, with extreme case assignment and a pattern-mixture analysis (Section 3.8). None of these analyses altered the conclusions. The baseline comparison is nevertheless limited by sparse documentation in the excluded group and cannot exclude differences in characteristics that were never recorded. The analytical cohort is best interpreted as representative of patients with complete six-month ascertainment rather than of all operated patients.

The cohort is also small in relation to what was asked of it. Seventy-eight patients is modest for prediction model development. Applying the criteria of Riley et al. [34,35] post hoc, with an outcome proportion of 0.641 and each model evaluated against its own cross-validated C-statistic (0.903 for the three-predictor model, 0.881 for the five-predictor set), the present cohort satisfies both the shrinkage and the small optimism criteria for the three-predictor model (47 and 62 participants required) but not for the five-predictor set (90 and 106 required), and it falls well short of the criterion for precise estimation of the overall outcome risk, which requires 354 participants irrespective of the number of predictors. The AUC confidence intervals are correspondingly wide (about ±0.086 across models). The events per variable ratio of the full multivariable model was low (EPV = 2.5, 28 events for 11 estimated coefficients), which is why the pre-specified three-predictor model (EPV ≈ 9) was designated the primary inferential result rather than the full model. No external validation was performed. Every performance estimate reported here, including the decision curve analysis, is internal to a single cohort of 78 patients, and internally cross-validated discrimination is known to be optimistic relative to performance in a new setting.

Three gaps in the recorded data deserve mention, of which the first is in our view the most consequential. Anticoagulant and antiplatelet use were not systematically documented. In a disease whose incidence is rising largely because of the growing use of these agents in an aging population, their absence removes a predictor of established clinical importance from every model reported here, and the systematic prospective recording of antithrombotic exposure is the single most important addition required of any future study. Hematoma volume was not computed; only maximum 2D width was available. Cortical atrophy was graded on a four-point visual scale by a single reader. Thus, neither inter-rater nor intra-rater reliability could be estimated, and misclassification of this predictor cannot be excluded. The grade was also unavailable for all but one of the excluded patients, so it could not enter the comparison of included with excluded patients. Preoperative midline shift showed no association with six-month GOS (per-mm OR = 1.02, 95% CI 0.94–1.10, *p* = 0.680) and was not entered into any multivariable model. Comorbidity was recorded but not used. Although alcoholism (17.9%), diabetes mellitus (15.4%), cardiac disease (28.2%), and hepatic disease (7.7%) were all documented, no comorbidity index was computed, and these variables entered none of the reported models. Thus, the competing cause argument advanced below rests on age and KPS rather than on a measured comorbidity burden. Missingness was also low only among the five machine learning predictors. Drainage was undocumented in 20 of 78 patients (25.6%) yet was imputed and retained in the exploratory full model. Thus, its coefficient rests substantially on imputed values.

The handling of missing data carries its own caveats. The missing at random assumption underlying multiple imputation was not formally tested, and the imputation used for the machine learning models was fitted once on the full analytical dataset—including the outcome—before cross-validation rather than separately within each training fold. Missingness within the analytical cohort was low (0–2.6% across the five predictors), which bounds the size of any such bias, but a small optimistic bias in the cross-validated discrimination estimates cannot be excluded. Thus, fold internal imputation is recommended for future confirmatory work. A second departure from full fold internality should be acknowledged alongside it. The five-predictor feature set was chosen after univariate screening on the complete analytical cohort rather than inside each training fold, so predictor selection was not fold internal either. Nor was any adjustment for multiplicity applied. Ten candidate predictors were screened univariately, and the nominal associations of cortical atrophy grade (*p* = 0.033) and age (*p* = 0.047) survive neither a Bonferroni correction (*p* = 0.333 and *p* = 0.469) nor a false discovery rate adjustment (*p* = 0.083 and *p* = 0.094) across that screen. The fifteen pairwise DeLong comparisons are likewise unadjusted, although none approached significance in any case.

Three further methodological limitations concern the models themselves. Calibration was summarized chiefly by the Brier score and by the calibration slope. No calibration plot is reported, so systematic over- or under-estimation of risk across parts of the probability range cannot be excluded. In addition, the slope of the five-predictor logistic classifier (0.69, 95% CI 0.38–0.99) indicates measurable over-fitting. The coefficients in Table 4 are apparent estimates to which no uniform shrinkage factor was applied. With the exception of the CART complexity parameter, hyperparameters were fixed a priori and not tuned, so the absence of any advantage for the ensemble and kernel methods may partly reflect untuned configurations rather than a genuine ceiling on achievable discrimination. Finally, the proportional odds assumption of the complementary ordinal model was not formally tested, and two of its five outcome categories contain fewer than eight patients.

The cohort is also heterogeneous in its management. Three index strategies were represented (burr hole craniostomy in 56 patients, craniotomy in 10, and initial conservative management followed by drainage in 12); no twist drill procedure was performed. The analyses were adjusted neither for management strategy nor for operating surgeon, and a cohort of this size cannot support such adjustment. The secondary analysis of recurrence is subject to the same constraint. With 14 events, no multivariable model of recurrence was feasible, and the near significant association of atrophy grade with re-operation reported in Section 3.9 must be regarded as hypothesis generating.

Finally, the outcome itself is imperfectly captured. The six-month GOS was assigned by the treating neurosurgeon at routine follow-up; it was neither blinded to the baseline clinical and imaging findings nor centrally adjudicated. Non-differential misclassification would attenuate the associations reported here, whereas an assessor aware of a poor preoperative Karnofsky score could plausibly bias the KPS–outcome association away from the null. The same concern applies to the exposure. Cortical atrophy was graded retrospectively by a single reader who was not blinded to outcome, and unblinded grading of a subjectively scored predictor can bias its association in either direction. Dichotomizing GOS at 4–5 versus 1–3 discards clinically meaningful gradation, lumping death (GOS 1) with severe disability (GOS 3) and good recovery (GOS 5) with moderate disability (GOS 4). The ordinal proportional odds model (Supplementary Table S3), which preserves the full scale, confirmed the KPS effect and, like the dichotomized models, showed no independent effect of cortical atrophy grade; it is reported as a complementary analysis. Of the 19 deaths at six months, 15 occurred after discharge, and no formal competing-risks analysis was performed. In a cohort of this age, with a mean of 69.1 years and frequent comorbidity, an unfavorable six-month GOS will in some patients reflect the natural history of coexisting disease rather than the hematoma or its treatment. The endpoint used here is all-cause functional status and cannot separate the two; cause-specific outcome adjudication would be required to do so. Follow-up was limited to six months. A more fundamental caveat should be stated plainly: preoperative KPS and the GOS endpoint are both clinician-rated ordinal measures of global function, separated only by six months. The dominance of KPS in every model is therefore partly definitional rather than mechanistic, and these models are better described as quantifying the persistence of functional status than as identifying modifiable determinants of recovery.

## 5. Conclusions

In this methodologically revised analysis of the 78 of 101 registry patients with ascertainable six-month outcomes, using multiple imputation and repeated held-out cross-validation, a three-predictor logistic model comprising preoperative Karnofsky Performance Status, cortical atrophy grade, and maximum hematoma width identifies preoperative KPS as the single independent prognostic factor for six-month functional outcome in CSDH (OR = 1.14 per point, 95% CI 1.07–1.20, *p* < 0.001). Cortical atrophy grade was associated with outcome on univariate analysis (OR = 0.57 per grade, *p* = 0.033) but not after adjustment for KPS (OR = 1.08, 95% CI 0.53–2.22, *p* = 0.833). The adjusted interval is wide enough to contain the unadjusted estimate, so these data exclude neither a moderate protective effect nor none at all. The reading of atrophy as a marker of premorbid frailty is a hypothesis since no direct measure of frailty was available. Atrophy grade was, however, the variable most nearly associated with re-operation (OR = 1.95 per grade, *p* = 0.058), which may be the more productive line of inquiry. Six machine learning classifiers trained on five core preoperative features achieved held-out cross-validated discrimination in the 0.870–0.885 AUC range, with XGBoost achieving the numerically highest discrimination (AUC = 0.885) and the lowest Brier score (0.120). No between-model AUC difference was statistically significant (all DeLong *p* ≥ 0.123), and the whole observed spread of 0.015 was well below the 0.031 difference this cohort had power to detect, so this is an absence of evidence rather than evidence of equivalence. All performance estimates are derived exclusively from out of fold predictions, which removes model-fitting optimism, although a small residual bias from imputing missing predictors on the full dataset before cross-validation cannot be fully excluded. No machine learning classifier outperformed logistic regression by a statistically significant margin. The three-predictor model, put through the identical cross-validation pipeline, reached AUC 0.903 (95% CI 0.833–0.972) with a calibration slope consistent with unity, so a simple, interpretable three-predictor logistic score is the preferred practical tool on the present evidence. Its coefficients are apparent estimates and require recalibration before external use. Because most of the discrimination is attributable to preoperative KPS—a measure conceptually close to the outcome—and removing it costs 0.10 to 0.16 in AUC, these models should be regarded as exploratory. Two further caveats belong with that judgement: the cohort falls well short of the 354 participants the Riley criteria require for precise estimation of the overall outcome risk, and antithrombotic exposure, the most consequential gap in the recorded data, entered none of the models. One observation could not be quantified at all: none of the nine patients with a documented coagulopathy achieved a favorable outcome, a zero cell that makes the effect non-estimable rather than absent and that warrants targeted study in a larger cohort. Prospective, multicenter studies with larger cohorts, contemporary treatment protocols (including middle meningeal artery embolization) and pre-registered external validation are required to confirm these predictors and to establish clinical utility.

## Figures and Tables

**Table 1:** Baseline characteristics of the analytical cohort (*n* = 78) stratified by six-month functional outcome.

Variable	Overall (*n* = 78)	Favorable (*n* = 50)	Unfavorable (*n* = 28)
Age, years (mean ± SD)	69.1 ± 11.4	67.2 ± 11.8	72.6 ± 10.0
Sex, male, *n* (%)	52 (66.7)	39 (78.0)	13 (46.4)
GCS on admission (median, IQR)	14 (12–15)	14 (14–15)	12 (8–14)
Preoperative KPS (mean ± SD)	52.2 ± 20.3	62.4 ± 15.5	34.3 ± 14.5
Maximum hematoma width, mm	22.1 ± 8.0	22.4 ± 8.2	21.4 ± 7.7
Bilateral hematoma, *n* (%)	28 (35.9)	17 (34.0)	11 (39.3)
Cortical atrophy grade (median, IQR)	2 (1–3)	2 (1–2)	2 (2–3)
Coagulopathy, *n* (%)	9 (11.5)	0 (0.0)	9 (32.1)
Drainage used, *n* (%)	56 (71.8)	36 (72.0)	20 (71.4)
Recurrence, *n* (%)	14 (17.9)	6 (12.0)	8 (28.6)
In-hospital mortality, *n* (%)	4 (5.1)	0 (0.0)	4 (14.3)
CT density, *n* (%)			
Hyperdense	1 (1.3)	0 (0.0)	1 (3.6)
Mixed	62 (79.5)	41 (82.0)	21 (75.0)
Isodense	5 (6.4)	4 (8.0)	1 (3.6)
Hypodense	10 (12.8)	5 (10.0)	5 (17.9)
Preop MSL, mm (mean ± SD)	8.3 ± 5.8	8.5 ± 5.9	7.9 ± 5.9

Cortical atrophy grade distribution (overall/favorable/unfavorable): grade 0, 11 (14.1%)/9 (18.0%)/2 (7.1%); grade 1, 18 (23.1%)/13 (26.0%)/5 (17.9%); grade 2, 29 (37.2%)/19 (38.0%)/10 (35.7%); and grade 3, 20 (25.6%)/9 (18.0%)/11 (39.3%). The grade was available for all 78 patients. Percentages use the column total as denominator, so rows with missing values do not sum to 100%. Drainage was documented for 58 of the 78 patients (56 of 58, 96.6%, received a drain) and in-hospital mortality for 77. GCS = Glasgow Coma Scale; KPS = Karnofsky Performance Score; SD = standard deviation; IQR = interquartile range.

**Table 2:** Logistic regression results pooled across m = 20 imputations using Rubin’s rules (*n* = 78). (Upper panel): Exploratory full multivariable model estimated by Firth penalized likelihood. (Lower panel): Pre-specified parsimonious three-predictor model (EPV ≈ 9).

Predictor	OR	95% CI	*p*
(Upper panel) exploratory full model, Firth penalized likelihood (*n* = 78)			
Age (per year)	0.97	0.89–1.05	0.461
Male sex	2.88	0.60–13.77	0.185
GCS on admission	0.86	0.55–1.35	0.518
Preoperative KPS	1.11	1.02–1.20	0.011
Maximum hematoma width	1.01	0.91–1.12	0.923
Bilateral hematoma	1.05	0.24–4.63	0.953
Surgery: burr hole	1.28	0.008–210.87	0.924
Surgery: craniotomy	1.26	0.011–139.95	0.922
Drainage	1.31	0.012–142.04	0.909
Cortical atrophy grade (per grade)	0.84	0.37–1.88	0.668
Coagulopathy ‡	0.03	0.001–0.64	0.025
(Lower panel) parsimonious model (*n* = 78)			
Preoperative KPS	1.14	1.07–1.20	<0.001
Cortical atrophy grade (per grade)	1.08	0.53–2.22	0.833
Maximum hematoma width	1.08	0.98–1.19	0.126

Estimates in the upper panel were obtained using Firth penalized likelihood, which returns finite estimates despite the quasi-separation caused by the absence of favorable outcomes among patients with coagulopathy (0 of 9); this panel is exploratory and is not the basis of any inferential claim. Cortical atrophy was entered as the four-point visual grade. ‡ The penalized estimate for coagulopathy is determined entirely by that zero cell and is not interpretable as an effect size; it is therefore omitted from Figure 3. OR = odds ratio; CI = confidence interval; GCS = Glasgow Coma Scale; KPS = Karnofsky Performance Score.

**Table 3:** Performance metrics of six machine learning classifiers evaluated using stratified 10-fold repeated cross-validation (5 repeats); all metrics computed from held-out fold predictions on the parsimonious five-predictor feature set.

Model	AUC (95% CI)	Sens	Spec	Acc	Brier
XGBoost	0.885 (0.80–0.97)	0.86	0.79	0.833	0.120
Logistic regression	0.881 (0.80–0.96)	0.88	0.75	0.833	0.136
Neural network	0.879 (0.79–0.96)	0.86	0.75	0.821	0.125
SVM	0.879 (0.79–0.96)	0.88	0.64	0.795	0.135
Decision tree	0.871 (0.78–0.96)	0.80	0.89	0.833	0.121
Random forest	0.870 (0.78–0.96)	0.88	0.71	0.821	0.130

AUC = area under the ROC curve (DeLong 95% CI); Sens = sensitivity; Spec = specificity; Acc = accuracy; Brier = Brier score (lower = better calibration). Positive and negative predictive values (PPV, NPV) are reported in Supplementary Table S2. XGBoost showed the best balance of discrimination and calibration. No pairwise DeLong test reached significance; the smallest *p* value was 0.123 for XGBoost versus random forest.

**Table 4:** Pooled coefficients of the parsimonious three-predictor logistic model (*n* = 78; m = 20 imputations, Rubin’s rules), on the log-odds scale. These are the estimates underlying the odds ratios in the lower panel of Table 2, reported so that the model can be applied to an individual patient and validated externally.

Term	β (log-odds)	SE	OR (95% CI)	*p*
Intercept	−7.5398	2.2572	—	<0.001
Preoperative KPS (per point)	0.1272	0.0292	1.14 (1.07–1.20)	<0.001
Cortical atrophy grade (0–3)	0.0776	0.3669	1.08 (0.53–2.22)	0.833
Maximum hematoma width (per mm)	0.0768	0.0501	1.08 (0.98–1.19)	0.126

KPS = Karnofsky Performance Score; SE = standard error; OR = odds ratio; CI = confidence interval. The predicted probability of a favorable six-month outcome (GOS 4–5) is obtained as *p* = 1/(1 + exp(−L)), where L = −7.5398 + 0.1272 × KPS + 0.0776 × (cortical atrophy grade, 0 to 3) + 0.0768 × (maximum hematoma width in mm). A patient with a preoperative KPS of 60, atrophy grade 0, and a 22 mm hematoma has L = 1.78 and a predicted probability of 0.86. A patient with a KPS of 40, atrophy grade 3, and the same hematoma width has L = −0.53 and a predicted probability of 0.37. Only the KPS coefficient differed significantly from zero. Because these estimates derive from a single small cohort, they require external validation before clinical use.

## Data Availability

The data presented in this study are available on reasonable request from the corresponding author. The data are not publicly available because they are derived from identifiable hospital records and their release is restricted by the institutional data protection policy of the University Clinical Centre of Vojvodina. The full reproducible analysis pipeline is available from the corresponding author.

## Supplementary Materials

The following supporting information can be downloaded at https://www.mdpi.com/article/10.3390/jcm15176822/s1.
