# Supplementary material for: Predictors of Six-Month Functional Outcome After Chronic Subdural Hematoma Surgery: Logistic Regression Versus Machine Learning
Source: J Clin Med. 2026 Sep 3;15(17):6822. doi: 10.3390/jcm15176822 (PMC13565903; doi:10.3390/jcm15176822)
Supplement: Supplementary file 1 [file jcm-15-06822-s001.zip › jcm-4506923-supplementary.pdf]

Supplementary Materials

Title: Predictors of Six-Month Functional Outcome After Chronic Subdural Hematoma Surgery: Logistic Regression Versus Machine Learning

Authors: Mirela Juković, Srdjan Stošić, Jagoš Golubović, Slobodan Pantelinac, Dejan B. Stojanović

Table S1. Missingness by predictor in the analytical cohort (n = 78). Counts are the number of patients with an observed and with a missing value for each predictor; percentages are computed on the 78 patients retained in the analysis. Missing values were handled by multiple imputation (m = 20) for the regression analyses; for the machine-learning analyses a single MICE imputation was used, as described in Section 2.5.

Predictor	Present	Missing	% missing
Age	78	0	0.0
Preoperative GCS	77	1	1.3
Preoperative KPS	77	1	1.3
Maximum hematoma width	76	2	2.6
Cortical atrophy grade	78	0	0.0

GCS = Glasgow Coma Scale; KPS = Karnofsky Performance Status.

Table S2. Full discrimination and calibration metrics of the six classifiers trained on the five preoperative predictors, ordered by decreasing AUC. Confidence intervals for the AUC are DeLong intervals; all metrics are out-of-fold estimates.

Model	AUC (95% CI)	Sens	Spec	PPV	NPV	Accuracy	Brier
XGBoost	0.885 (0.798–0.972)	0.86	0.79	0.88	0.76	0.83	0.120
Logistic Regression	0.881 (0.803–0.960)	0.88	0.75	0.86	0.78	0.83	0.136
Neural Network	0.879 (0.795–0.964)	0.86	0.75	0.86	0.75	0.82	0.125
SVM	0.879 (0.794–0.964)	0.88	0.64	0.81	0.75	0.79	0.135
Decision Tree	0.871 (0.779–0.964)	0.80	0.89	0.93	0.71	0.83	0.121
Random Forest	0.870 (0.779–0.961)	0.88	0.71	0.85	0.77	0.82	0.130

AUC = area under the receiver-operating-characteristic curve; CI = confidence interval; Sens = sensitivity; Spec = specificity; PPV = positive predictive value; NPV = negative predictive value; Brier = Brier score (lower is better).

Table S3. Ordinal proportional-odds model for the full six-month Glasgow Outcome Scale (GOS 1–5), parsimonious predictor set, fitted across the 20 imputed datasets. Odds ratios above 1 indicate higher odds of a better GOS category.

Predictor	OR	95% CI	p
Preoperative KPS (per point)	1.111	1.071–1.153	<0.001
Cortical atrophy grade (per grade)	0.942	0.563–1.576	0.819
Maximum hematoma width (per mm)	1.060	0.991–1.133	0.089

CI = confidence interval; GOS = Glasgow Outcome Scale; KPS = Karnofsky Performance Status; OR = odds ratio.

Table S4. KPS-excluded sensitivity analysis. All six classifiers were re-trained after removing preoperative KPS from the predictor set, ordered by decreasing AUC.

Model	AUC (95% CI)	Sens	Spec	PPV	NPV	Accuracy	Brier
Logistic Regression	0.779 (0.662–0.895)	0.90	0.54	0.78	0.75	0.77	0.168
Neural Network	0.746 (0.620–0.873)	0.88	0.57	0.79	0.73	0.77	0.178
SVM	0.729 (0.600–0.857)	0.90	0.39	0.73	0.69	0.72	0.188
XGBoost	0.726 (0.595–0.857)	0.90	0.54	0.78	0.75	0.77	0.182
Random Forest	0.723 (0.593–0.852)	0.88	0.54	0.77	0.71	0.76	0.184
Decision Tree	0.719 (0.592–0.846)	0.84	0.50	0.75	0.64	0.72	0.197

AUC = area under the receiver-operating-characteristic curve; CI = confidence interval; KPS = Karnofsky Performance Status; Sens = sensitivity; Spec = specificity; PPV = positive predictive value; NPV = negative predictive value; Brier = Brier score (lower is better).

Table S5. Comparison of the 78 patients included in the analytical cohort and the 23 excluded patients (16 with no six-month outcome recorded, 7 with GOS coding that could not be verified). Variables recorded after admission (length of stay, postoperative midline shift, recurrence, mortality) are included alongside the baseline variables. Continuous variables were compared with the Wilcoxon rank-sum test and binary variables with Fisher's exact test; the last column gives the number of missing values in the included and in the excluded group.

Variable	Included (n = 78)	Excluded (n = 23)	p	Missing, incl./excl.
Age, years (mean ± SD)	69.1 ± 11.4	72.6 ± 14.5	0.124	0 / 2
GCS on admission (mean ± SD)	12.8 ± 2.9	14.1 ± 1.4	0.158	1 / 14
Preoperative KPS (mean ± SD)	52.2 ± 20.3	64.3 ± 17.2	0.161	1 / 16
Maximum hematoma width, mm	22.1 ± 8.0	25.6 ± 6.6	0.219	2 / 15
Postoperative midline shift, mm	4.5 ± 4.2	2.7 ± 3.5	0.172	2 / 10
Length of stay, days	10.6 ± 7.1	7.4 ± 3.3	0.191	1 / 14
Cortical atrophy grade	1.7 ± 1.0	2.0 ± NA	NA	0 / 22
Male sex, n (%)	52/78 (66.7)	13/23 (56.5)	0.459	0 / 0
Bilateral hematoma, n (%)	28/78 (35.9)	5/22 (22.7)	0.310	0 / 1
Any cortical atrophy (grade ≥ 1), n (%)	67/78 (85.9)	1/1 (100.0)	NA	0 / 22
Coagulopathy, n (%)	9/78 (11.5)	2/8 (25.0)	0.271	0 / 15
Subdural drainage, n (%)	56/58 (96.6)	8/11 (72.7)	0.026	20 / 12
Alcoholism, n (%)	14/78 (17.9)	1/8 (12.5)	1.000	0 / 15
Diabetes mellitus, n (%)	12/78 (15.4)	1/8 (12.5)	1.000	0 / 15
Heart disease, n (%)	22/78 (28.2)	1/8 (12.5)	0.676	0 / 15
Liver disease, n (%)	6/78 (7.7)	0/8 (0.0)	1.000	0 / 15
Hydrocephalus, n (%)	2/77 (2.6)	1/8 (12.5)	0.259	1 / 15
Recurrence, n (%)	14/78 (17.9)	4/22 (18.2)	1.000	0 / 1
In-hospital mortality, n (%)	4/77 (5.2)	0/9 (0.0)	1.000	1 / 14

GCS = Glasgow Coma Scale; KPS = Karnofsky Performance Status; SD = standard deviation. NA = not estimable because of sparse data in the excluded group.

Table S6. Firth penalized-likelihood full multivariable logistic model for a favorable six-month outcome (n = 78; m = 20 imputations, pooled by Rubin's rules). Penalized likelihood was used because the unpenalized full model showed quasi-separation, no patient with coagulopathy having achieved a favorable outcome.

Predictor	β (log-odds)	SE	OR (95% CI)	p
Intercept	−1.036	3.832	0.355 (0.00019–648.746)	0.787
Age (per year)	−0.031	0.041	0.970 (0.894–1.052)	0.461
Male sex	1.058	0.798	2.880 (0.602–13.775)	0.185
Preoperative GCS (per point)	−0.149	0.231	0.861 (0.548–1.354)	0.518
Preoperative KPS (per point)	0.101	0.040	1.106 (1.023–1.196)	0.011
Maximum hematoma width (per mm)	0.005	0.053	1.005 (0.905–1.116)	0.923
Surgery: burr-hole	0.247	2.596	1.280 (0.008–210.868)	0.924
Surgery: craniotomy	0.234	2.397	1.264 (0.011–139.954)	0.922
Bilateral hematoma	0.045	0.759	1.046 (0.236–4.633)	0.953
Subdural drainage	0.271	2.381	1.312 (0.012–142.039)	0.909
Cortical atrophy grade (per grade)	−0.177	0.413	0.838 (0.373–1.883)	0.668
Coagulopathy	−3.579	1.598	0.028 (0.001–0.639)	0.025

CI = confidence interval; GCS = Glasgow Coma Scale; KPS = Karnofsky Performance Status; OR = odds ratio; SE = standard error; β = regression coefficient on the log-odds scale.

Table S7. Ridge and lasso penalized logistic regression over the full predictor set, with the penalty selected by cross-validation within each of the 20 imputed datasets. The last column reports in how many of the 20 imputations the lasso coefficient was retained (non-zero).

Predictor	Ridge β	Ridge OR	Lasso β	Lasso OR	Non-zero in lasso (of 20)
Intercept	−2.2149	0.109	−3.5114	0.03	20
Age (per year)	−0.0203	0.98	0	1	0
Male sex	0.6081	1.837	0.3073	1.36	20
Preoperative GCS (per point)	0.116	1.123	0	1	0
Preoperative KPS (per point)	0.0514	1.053	0.0827	1.086	20
Maximum hematoma width (per mm)	0.0187	1.019	0.0019	1.002	4
Surgery: burr-hole	0.1766	1.193	0	1	0
Surgery: craniotomy	−0.0208	0.979	0	1	0
Bilateral hematoma	−0.173	0.841	0	1	0
Subdural drainage	−0.0242	0.976	0	1	0
Cortical atrophy grade (per grade)	−0.234	0.791	−0.0081	0.992	20
Coagulopathy	−2.7317	0.065	−2.4127	0.09	20

OR = odds ratio; β = penalized regression coefficient on the log-odds scale. Coefficients shown as 0 (OR = 1) were shrunk to exactly zero by the lasso in every imputation. Penalized coefficients are shown as reported by the fitting routine and are not accompanied by confidence intervals or p-values.

Table S8. Sensitivity of the parsimonious model to the 23 patients with a missing six-month outcome. The primary multiple-imputation analysis is compared with inverse-probability weighting, with the two extreme deterministic imputations, and with a pattern-mixture analysis in which the log-odds of a favorable outcome among the non-observed patients are shifted by δ.

Analysis	KPS OR (95% CI)	p	Atrophy grade OR (95% CI)	p	Width OR (95% CI)	p
Primary: multiple imputation, n = 78	1.136 (1.072–1.203)	<0.001	1.081 (0.526–2.218)	0.833	1.080 (0.979–1.191)	0.126
Inverse-probability weighting	1.140 (1.073–1.210)	<0.001	1.078 (0.519–2.242)	0.840	1.078 (0.970–1.197)	0.162
All 23 excluded set favorable	1.096 (1.045–1.151)	<0.001	1.206 (0.602–2.416)	0.597	1.061 (0.973–1.157)	0.177
All 23 excluded set unfavorable	1.067 (1.032–1.103)	<0.001	0.921 (0.513–1.651)	0.781	1.023 (0.955–1.096)	0.522
Pattern-mixture, δ = −3	1.104 (1.054–1.157)	<0.001	1.023 (0.523–1.999)	0.9477	1.039 (0.959–1.126)	0.3442
Pattern-mixture, δ = −2	1.123 (1.065–1.184)	<0.001	1.017 (0.525–1.971)	0.9605	1.056 (0.963–1.158)	0.2471
Pattern-mixture, δ = −1	1.129 (1.070–1.190)	<0.001	1.027 (0.501–2.106)	0.9415	1.062 (0.964–1.171)	0.2208
Pattern-mixture, δ = 0	1.135 (1.075–1.199)	<0.001	1.061 (0.517–2.179)	0.8709	1.074 (0.973–1.185)	0.1563
Pattern-mixture, δ = +1	1.135 (1.072–1.201)	<0.001	1.128 (0.560–2.272)	0.7369	1.081 (0.971–1.203)	0.1541
Pattern-mixture, δ = +2	1.124 (1.065–1.187)	<0.001	1.147 (0.550–2.392)	0.7144	1.079 (0.978–1.190)	0.1309

CI = confidence interval; KPS = Karnofsky Performance Status; OR = odds ratio; Width = maximum hematoma width. δ is the shift, on the log-odds scale, applied to the probability of a favorable outcome among the patients with a missing outcome.

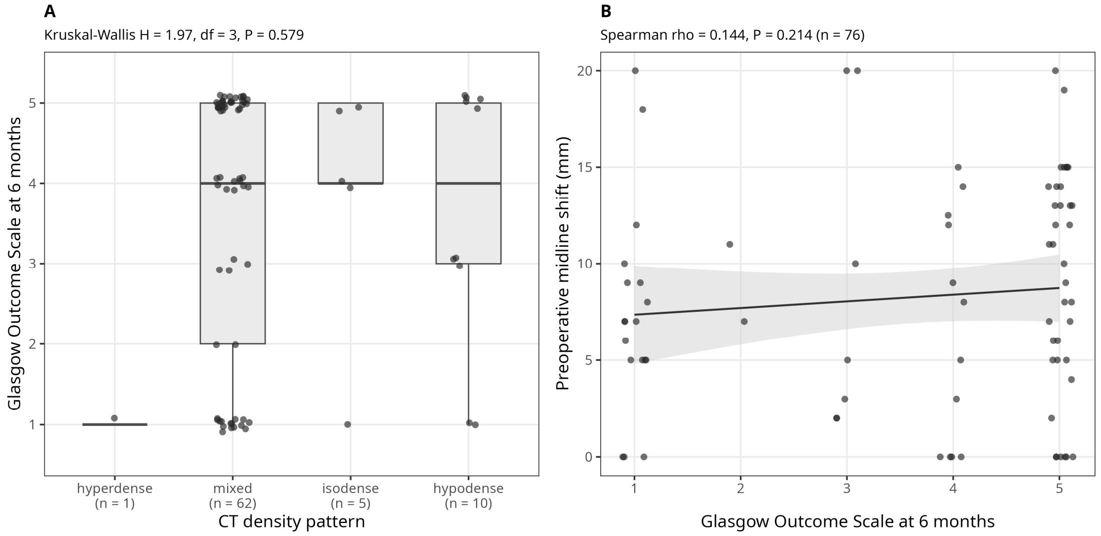

Figure S1. (A) Six-month Glasgow Outcome Scale by CT density pattern (n = 78; hyperdense 1, mixed 62, isodense 5, hypodense 10). Kruskal–Wallis H = 1.969, df = 3, p = 0.579. (B) Preoperative midline shift versus six-month GOS (n = 76); Spearman rho = 0.144, p = 0.214.

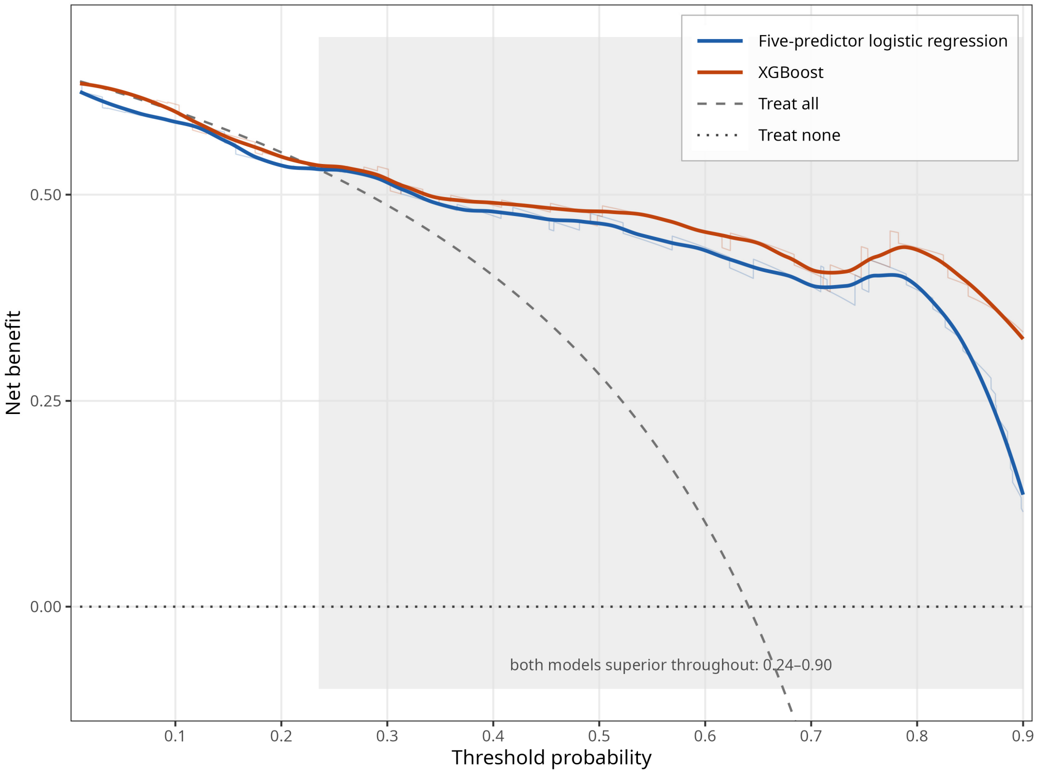

Figure S2. Decision-curve analysis of the out-of-fold predicted probabilities of the five-predictor logistic classifier and XGBoost, against the treat-all and treat-none reference strategies (outcome prevalence 0.641). Heavy lines are LOESS-smoothed (span 0.20) for legibility; the underlying empirical step functions are shown faintly behind them, and all reported values are taken from the empirical curves. Both models yield higher net benefit than either reference strategy across the whole range shown. The display is truncated at a threshold probability of 0.90 because beyond it a single discordant patient shifts net benefit by more than 0.11 (the weight $t/(1 - t)$ exceeds 9 in a cohort of 78), so the estimate is no longer interpretable: the empirical upper limit of the superior range is 0.93 but its bootstrap 95% interval is 0.87 to 0.98.
